# Nanozyme-Participated Biosensing of Pesticides and Cholinesterases: A Critical Review

**DOI:** 10.3390/bios11100382

**Published:** 2021-10-09

**Authors:** Hengjia Zhu, Peng Liu, Lizhang Xu, Xin Li, Panwang Hu, Bangxiang Liu, Jianming Pan, Fu Yang, Xiangheng Niu

**Affiliations:** 1 School of Agricultural Engineering, Jiangsu University, Zhenjiang 212013, China; 2112016003@stmail.ujs.edu.cn; 2 Institute of Green Chemistry and Chemical Technology, School of Chemistry and Chemical Engineering, Jiangsu University, Zhenjiang 212013, China; 2221912033@stmail.ujs.edu.cn (P.L.); 2111712012@stmail.ujs.edu.cn (X.L.); 2212112053@stmail.ujs.edu.cn (P.H.); 2212112038@stmail.ujs.edu.cn (B.L.); pjm@ujs.edu.cn (J.P.); 3 School of Environmental and Chemical Engineering, Jiangsu University of Science and Technology, Zhenjiang 212003, China; fuyang@just.edu.cn; 4 Key Laboratory of Functional Molecular Solids of Ministry of Education, Anhui Normal University, Wuhu 241002, China; 5 State Key Laboratory of Urban Water Resource and Environment, Harbin Institute of Technology, Harbin 150090, China

**Keywords:** pesticide, cholinesterase, nanozyme, sensor, biosensor

## Abstract

To improve the output and quality of agricultural products, pesticides are globally utilized as an efficient tool to protect crops from insects. However, given that most pesticides used are difficult to decompose, they inevitably remain in agricultural products and are further enriched into food chains and ecosystems, posing great threats to human health and the environment. Thus, developing efficient methods and tools to monitor pesticide residues and related biomarkers (acetylcholinesterase and butylcholinesterase) became quite significant. With the advantages of excellent stability, tailorable catalytic performance, low cost, and easy mass production, nanomaterials with enzyme-like properties (nanozymes) are extensively utilized in fields ranging from biomedicine to environmental remediation. Especially, with the catalytic nature to offer amplified signals for highly sensitive detection, nanozymes were finding potential applications in the sensing of various analytes, including pesticides and their biomarkers. To highlight the progress in this field, here the sensing principles of pesticides and cholinesterases based on nanozyme catalysis are definitively summarized, and emerging detection methods and technologies with the participation of nanozymes are critically discussed. Importantly, typical examples are introduced to reveal the promising use of nanozymes. Also, some challenges in the field and future trends are proposed, with the hope of inspiring more efforts to advance nanozyme-involved sensors for pesticides and cholinesterases.

## 1. Introduction

Pesticides, a class of important agricultural production materials, were widely used to control insects, grasses, and diseases harmful to crops. Safely and appropriately utilizing pesticides can effectively improve the output and quality of agricultural products for the growing population [1]. Nevertheless, every coin has its two sides. The misuse and abuse of pesticides became global issues, posing a serious threat to human health and the environment [2]. Most of pesticides used are difficult to degrade, leading to pesticide residues in the ecosystem for a long time. Pesticides get into the food chain through air, water, and soil, eventually accumulating in human body and animals and causing a series of problems [3,4,5]. Currently, organophosphorus pesticides (OPs) and carbamate pesticides (CPs) dominate a major market. They can irreversibly bind to and inactivate cholinesterases (ChE) including acetylcholinesterase (AChE) and butylcholinesterase (BChE) [6], which are critical enzymes affecting the nervous system. The inactivation of ChE is closely related to many diseases, such as Parkinson’s disease, Alzheimer’s disease, and Huntington’s disease [7,8,9,10,11]. Therefore, it is of great importance to monitor pesticides and their biomarkers for food safety and human health.

Up to now, a number of methods were established for the detection and quantification of pesticides and related biomarkers. Instrumental detection, such as gas chromatography, high-performance liquid chromatography, and capillary electrophoresis [12,13], is proven as an effective means to detect pesticides. Although these instrumental approaches present the merits of high selectivity and excellent repeatability, the limits including expensive equipment, time-consuming process, and complex operation seriously restrict their use for on-site rapid detection [14]. Contrastively, the enzyme inhibition principle is intensively explored for the biosensing of OPs and CPs [15,16]. With the attractive advantages of convenient operation and rapid response, there are numerous of colorimetric, fluorescence, and electrochemical pesticide sensors that were developed based on the principle [6,17,18]. However, the natural enzymes employed are very vulnerable in harsh environments, resulting in the poor robustness of these enzyme-based biosensors in practical applications [19]. Besides, the use of multiple tandem bioenzymes leads to the relatively high cost of these sensors. How to realize the sensing of pesticides and their biomarkers with robust performance and lower costs is an issue that still needs to be addressed.

Nanomaterials with enzyme-like catalytic characteristics, defined as ‘nanozymes’, are a new class of artificial enzymes that emerged along with the development of nanotechnology [20]. In comparison with natural bioenzymes, nanozymes show the advantages of low costs, easy large-scale production, excellent tolerance to harsh environments, high stability, and on-demand tailorable activity [21,22,23,24,25]. As a result, they become a strong competitor of corresponding bioenzymes used for the sensing of various species [26,27,28,29,30], including pesticides and related biomarkers [19,31,32,33]. At present, nanozymes explored for pesticide analysis can be divided to oxidoreductases and hydrolases. On the one hand, oxidoreductases commonly used in pesticide detection include oxidase and peroxidase, whose main function is to provide catalytic amplified signals by reacting with corresponding substrates. Typically, Fe_3_O_4_ magnetic nanoparticles (MNPs) with horseradish peroxidase (HRP)-like activity can catalyze the oxidation of 3,3′,5,5′-tetramethylbenzidine (TMB), 3,3′-diaminobenzidine (DAB), *o*-phenylenediamine (OPD), and 2,2-azobis(3-ethylbenzothiazolin-6-sulfonic acid) (ABTS) with the help of H_2_O_2_ to generate colored or/and fluorescent products [34,35]. On the other hand, phosphatase mimics can be employed as a class of hydrolases in nanozyme-based pesticide assays. In these systems, phosphatase-like nanozymes produce quantitative information through hydrolyzing OPs [36].

**Figure 1:**
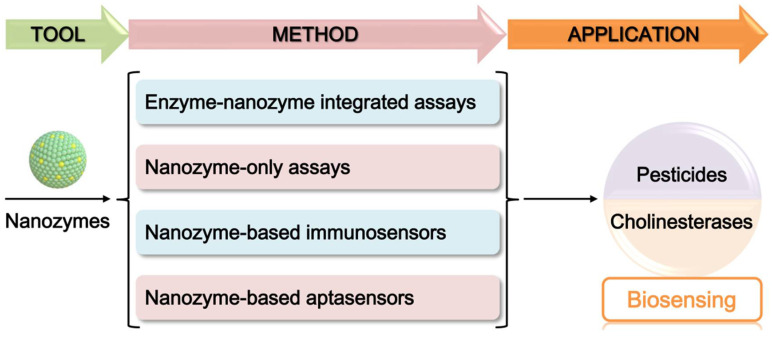
Schematic diagram of nanozymes as an emerging tool to develop various methods for biosensing of pesticides and cholinesterases.

With the above considerations, a series of methods and strategies were developed in recent years for pesticide and ChE biosensing by employing nanozymes as a tool to provide amplified signals, including enzyme-nanozyme integrated assays, nanozyme-only assays, nanozyme-based immunosensors, and nanozyme-based aptasensors (Figure 1). To highlight the progress in this field, here we intend to summarize the principles, methods, and applications of nanozyme-based sensors for pesticide and ChE detection. What should be stated is that although there are a number of thematic reviews on pesticide and related biomarker biosensing [15,37,38,39,40,41,42,43,44], no reviews focus on the emerging applications of nanozymes in pesticide biosensors. Our contribution is expected to fill in the gap. In this review, the principles and functions of nanozymes in the construction of pesticide sensors will be summarized. Typical examples are introduced to reveal the promising use of nanozymes in pesticide and ChE quantification, and the advantages and deficiencies of these methods are critically discussed. Finally, some challenges in this field and future trends are presented, with the hope of attracting more attention and interest to promote nanozyme-based sensors for pesticides and related biomarkers.

## 2. Enzyme–Nanozyme Integrated Assays

OPs and CPs can irreversibly inhibit the activity of some enzymes via the phosphorylation and carbamylation of their active centers, respectively [15,39]. According to such a mechanism, the enzyme inhibition principle was widely applied for the detection of pesticides and related biomarkers [45]. Owning to low costs, high stability, and robust catalytic performance, nanozymes can be combined with corresponding bioenzymes to establish enzyme–nanozyme integrated assays for pesticide and ChE determination.

### 2.1. Ternary ChE-CHO-Nanozyme Assays

**Figure 2:**
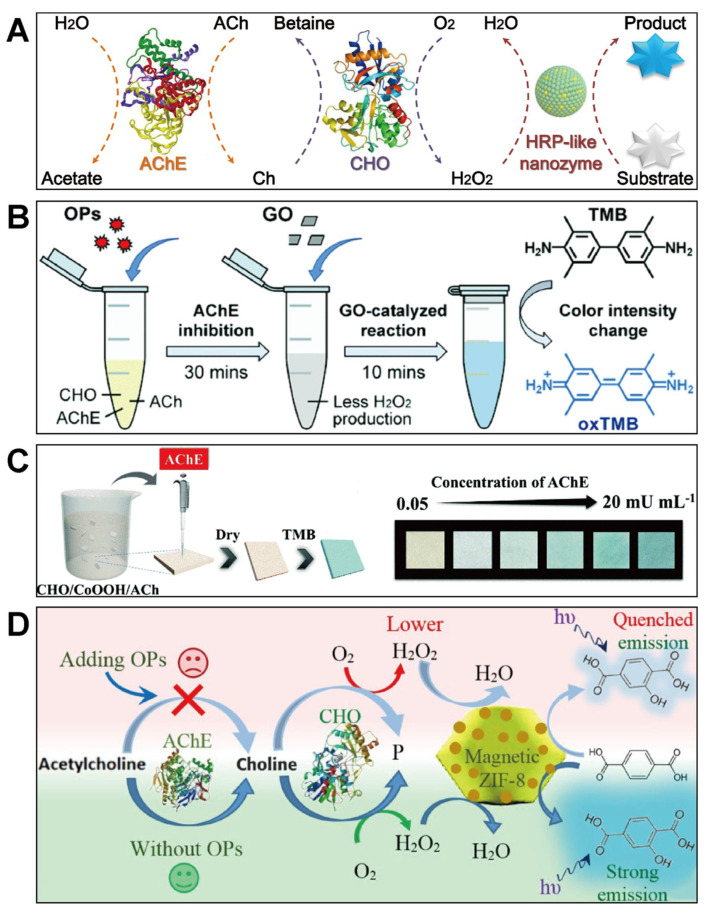
(**A**) illustrates detection principle of pesticides and cholinesterases based on ternary AChE−CHO−nanozyme cascade catalytic reactions; (**B**) shows colorimetric analysis of OPs via an AChE-CHO-GO cascade system (reprinted with permission from [49], Copyright 2020, Royal Society of Chemistry); (**C**) depicts fabrication of an AChE-CHO-CoOOH paper sensor for AChE activity determination (reprinted with permission from [50], Copyright 2019, Royal Society of Chemistry); (**D**) depicts fluorescence detection of OPs based on an AChE-CHO-nanozyme assay (reprinted with permission from [52], Copyright 2019, Elsevier).

Among enzyme–nanozyme integrated assays of pesticides and related biomarkers, a typical ternary cascade system consisting of two bioenzymes (ChE and choline oxidase (CHO)) and an HRP-like nanozyme was widely studied and applied [46,47,48,49,50,51,52]. The type of ChE mainly includes AChE and BChE. Here, taking AChE as an example, the principle of such an AChE-CHO-nanozyme detection platform is illustrated in Figure 2A. Briefly, AChE catalytically hydrolyzes the enzymatic substrate acetylcholine (ACh) to choline (Ch) and acetate first. Then, the produced Ch is oxidized under the catalysis of CHO to produce H_2_O_2_, which is a significant reactant for signal generation via an HRP-mimicking nanozyme catalyzing the chromogenic or/and fluorigenic reaction of H_2_O_2_ and corresponding substrates like TMB. In the presence of pesticides, the enzyme AChE is irreversibly inactivated, and the hydrolysis of ACh is interrupted. Eventually, the whole cascade reactions are terminated. As a result, the content of pesticide residues can be indirectly determined by such a ternary cascade system with different signal modes.

Attributed to the attractive features of easy readout and real-time visual detection via naked eyes, the colorimetric sensing mode attracted wide attention in the field of detecting pesticides and related biomarkers [37,38]. In a typical ChE-CHO-nanozyme colorimetric platform, chromogenic substrates including TMB, ABTS, and OPD are usually used to be catalyzed by an HRP-like nanozyme with the participation of H_2_O_2_ to produce color signals. A classic example was reported by Yan’s group, where a colorimetric assay composed of Fe_3_O_4_ MNPs with HRP-like activity, AChE and CHO was developed for OPs and nerve agent sensing [47]. Subsequently, a series of nanomaterials with peroxidase-like activity were explored for such a ternary colorimetric system [53,54,55,56,57]. Compared to that of natural HRP, all the artificial mimics are easier to get desired catalytic performance via purposeful activity modulation. For instance, Chu et al. synthesized size-tunable graphene oxides (GO) as an HRP mimic for the colorimetric detection of OPs (Figure 2B) [49]. In their study, when the enzyme AChE was inhibited by OPs, less H_2_O_2_ was produced. As a result, the H_2_O_2_-triggered chromogenic reaction of TMB under the peroxidase-like catalysis of small-sized GO was hindered, and a visual decrease of the color intensity could be observed for pesticide quantification. Another interesting example was based on single-atom nanozymes (SAN), whose active sites were atomically dispersed with ultrahigh catalytic activity [58,59]. Zhu’s group prepared a peroxidase-like SAN with high-concentration Cu sites on carbon nanosheets and constructed an AChE-CHO-SAN cascade reaction system for the colorimetric detection of ACh and OPs [48].

Although these ChE-CHO-nanozyme colorimetric assays exhibit a variety of merits and were widely applied for pesticide determination, most of them are performed in solution, which are inefficient and complex to operate for in-field detection. To simplify the procedure and enhance the convenience of such a ternary system, portable devices can be designed for facile colorimetric detection, such as paper-based biosensors combining with smartphones [60,61]. Typically, Jin et al. fabricated a test strip for convenient AChE detection through fixing the CHO/CoOOH/ACh mixture onto an absorbent paper (Figure 2C) [50]. With the presence of AChE, ACh loaded on the paper was catalyzed to produce H_2_O_2_, triggering the oxidation of the TMB substrate added to blue TMBox, and the color information could be read and analyzed by a smartphone for quantitative determination. Owning to convenient operation and fast response, paper-based biosensors are more suitable for the on-site detection of OPs and AChE. However, most of these reported devices are based on the colorimetric method by using physical interactions to fix the ternary ChE-CHO-nanozyme mixture onto a matrix. Both nonuniform distribution of these sensing elements and the color development difference caused by nonuniform mass transfer can affect the accuracy of these devices and their applications.

Apart from colorimetric assays, some other single-signal modes were also applied in the ChE-CHO-nanozyme system. Typically, fluorescence assays are more sensitive and less affected by the background color of matrices [62]. Similar to the colorimetric mode, fluorescence signals in the ChE-CHO-nanozyme platform are generated and amplified by the peroxidase mimic-catalyzed oxidation of corresponding substrates like OPD. Bagheri et al. designed a peroxidase-mimetic Fe_3_O_4_ NPs@ZIF-8 composite, which was able to catalyze the oxidation of terephthalic acid (TA) to produce a fluorescent product (2-hydroxy terephthalic acid, 2-HTA) in the participation of H_2_O_2_ [52]. On this basis, a label-free fluorescence biosensor for toxic OPs was established (Figure 2D). Different from traditional fluorescence sensors based on fluorescent probes or quantum dots [63,64], in the ChE-CHO-nanozyme system the fluorescence intensity is proportional to the content of H_2_O_2_ produced, and such that only the target-mediated fluorescence quenching model is explored and applied for pesticide biosensors. Besides, He et al. synthesized Fe-based metal–organic gel supported gold nanoparticles (AuNPs/MOG(Fe)) with excellent peroxidase-like activity through an in situ growth strategy [51], and they further fabricated a luminol-AuNPs/MOG(Fe)-H_2_O_2_ chemiluminescence platform for the quantitative analysis of pesticides. In the system, H_2_O_2_ was generated by the cascade catalysis of AChE and CHO, which could be transformed into multiple radicals inducing a chemiluminescence reaction for quantitative analysis. In addition to these nanozyme-participated single-signal modes for pesticide and related biomarker detection, a ChE-CHO-HRP mimic sensor based on an interesting double-signal mode was also developed [65].

In the modes mentioned above, some common rules should be followed. Firstly, the selectivity of these systems highly depends on the enzyme ChE, which acts as a recognition unit through the irreversible interaction between OPs and ChE. Secondly, with the involvement of CHO, the production of H_2_O_2_ is inevitable, so nanozymes with only peroxidase-like activity are ideally suitable. Undoubtedly, these rules pose some challenges to such a ChE-CHO-nanozyme system: on the one hand, due to the inherent fragile nature of bioenzymes, a mild detection environment is still necessary for these assays; on the other hand, many nanozymes often show multiple enzyme activities [66,67]. It is difficult to achieve a nanozyme that only presents peroxidase-mimicking activity [68], and such that interference originating from other enzyme-like activities may exist during the detection process.

### 2.2. Binary ChE-Nanozyme Assays

Although the ternary cascade system composed of ChE, CHO, and a peroxidase-like nanozyme can provide amplified signals, the low-efficiency multistep cascade reactions often result in poor sensitivity. If some bioenzymes are removed from the ternary system, the detection efficiency may be effectively increased. Given that pesticide residues show the inhibition impact on only ChE, the use of CHO can be avoided theoretically. Instead, efficient sensing platforms consisting of ChE and nanozymes can be explored to analyze pesticide residues.

#### 2.2.1. Generation of Thiol-Containing Compounds

**Figure 3:**
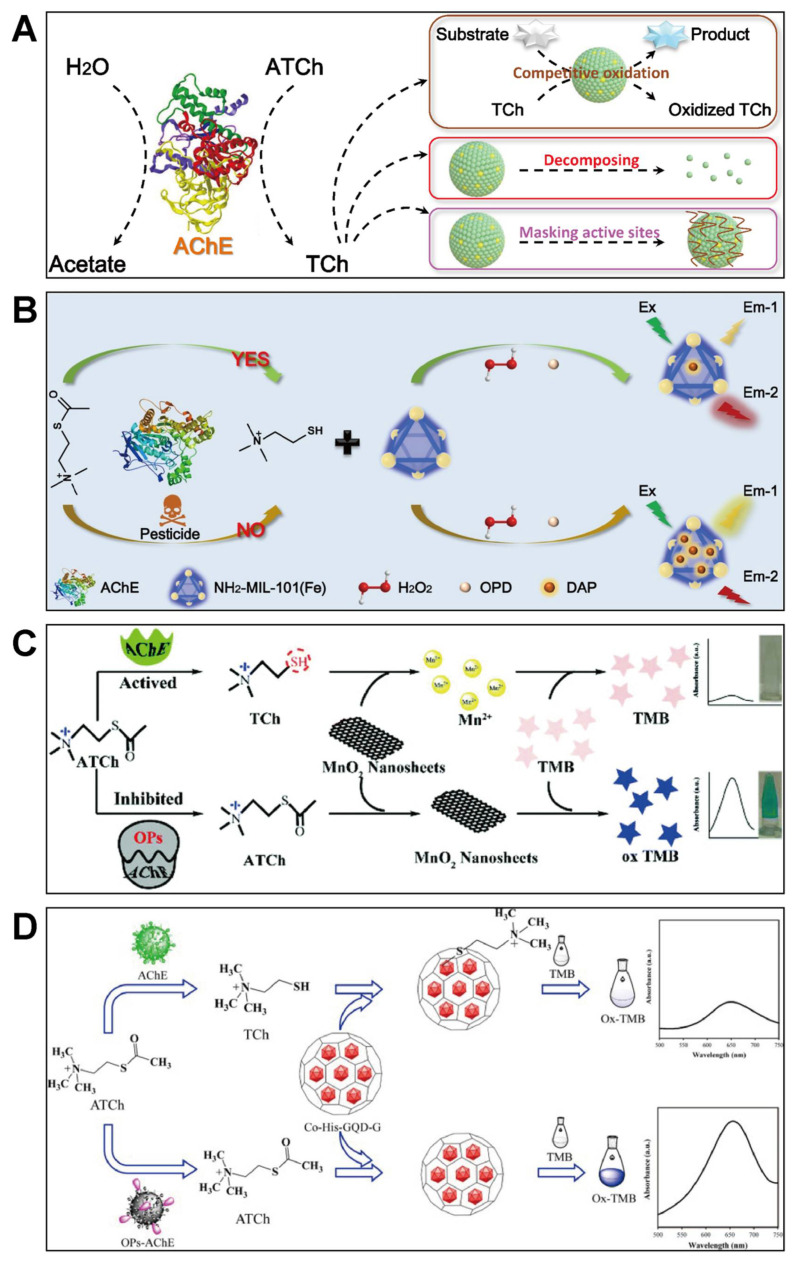
(**A**) explains possible effects of TCh generated from AChE catalysis on nanozyme sensing systems; (**B**) presents design of multifunctional NH_2_-MIL-101(Fe) for the ratiometric fluorescence determination of pesticides (reprinted with permission from [31], Copyright 2021, Elsevier); (**C**) illustrates biosensing of OPs via generated TCh decomposing oxidase-like MnO_2_ nanosheets (reprinted with permission from [72], Copyright 2009, Royal Society of Chemistry); (**D**) shows that TCh generated from AChE catalysis can mask active sites of Co-His-GOD-G for OPs analysis (reprinted with permission from [73], Copyright 2021, Elsevier).

In binary ChE-nanozyme assays, the nanozymes used are not limited to peroxidase mimics anymore. Some hydrolyzates originating from ChE catalysis can directly react with nanozymes to generate signals. Typically, acetylthiocholine (ATCh) is utilized as an enzymatic substrate to be hydrolyzed by AChE to thiocholine (TCh) and acetate (Figure 3A) [69]. The product TCh with a sulfhydryl group shows some unique characters [70,71]. It can be competitively oxidized under the catalysis of nanozymes. Also, it is able to decompose some nanozymes and mask their active sites, thus making impacts on nanozyme-catalyzed reactions. Table 1 summarizes the nanozyme-involved detection of pesticides and ChE based on different mechanisms caused by the generation of thiol-containing compounds.

In most of ChE-nanozyme assays for pesticides, the amplification of signals is caused by the catalytic oxidation of colored or/and fluorescent substrates. The species TCh produced from ATCh hydrolysis can make some impacts on the catalytic amplification of signals via a competitive reaction. Zhou’s group developed a colorimetric assay for the detection of OPs based on polyacrylic acid-coated cerium oxide nanoparticles (PAA-CeO_2_) as an oxidase mimic and AChE [74]. When incubating with OPs, the production of TCh would be hindered, and the competition effect was weakened. As a result, more TMB was catalytically oxidized by PAA-CeO_2_ to exhibit a deeper blue color. The ChE-nanozyme cascade system can also be designed on the basis of the TCh competition effect disturbing fluorescence resonance energy transfer or inner filter effect [81,82]. For instance, our group prepared bifunctional Fe-based metal–organic frameworks (NH_2_-MIL-101(Fe)) with both peroxidase-mimetic catalytic activity and photoluminescence for the ratiometric fluorescence sensing of carbaryl (Figure 3B) [31]. In such an assay, the produced TCh would inhibit the oxidation of OPD to fluorescent diaminophenazine (DPA), disturbing the inner filter effect between photoluminescent NH_2_-MIL-101(Fe) and DPA. In addition to the above direct competitive oxidation, the produced TCh can suppress the chromogenic process via re-reducing blue TMBox to colorless TMB [89,90], which also provides the basis for ChE and pesticide sensing based on nanozyme catalysis.

Different from the competitive oxidation between TCh and nanozyme substrates, TCh can also result in the degradation of some nanozymes directly. In the decomposition mechanism nanozymes employed to fabricate the ChE-nanozyme system are mainly Mn-based nanomaterials, such as MnO_2_ nanosheets and γ-MnOOH nanowires, both of which are easily decomposed by reducing agents including TCh, ascorbic acid, and glutathione [84,91,92,93]. In these reports, TCh generated by AChE and ATCh can efficiently decompose MnO_2_ and MnOOH to Mn^2+^ ions. As a result, the catalytic activity of these nanozymes loses, and the TMB oxidation process is suspended. Based on the above mechanism, Yan et al. explored oxidase-like ultrathin MnO_2_ nanosheets for the colorimetric sensing of AChE activity with TMB as a chromogenic substrate (Figure 3C) [72]. Mn-based nanomaterials often exhibit both oxidase- and peroxidase-like activities as well as a dark color, producing interference for these ChE-nanozyme colorimetric assays. To avoid this issue, Wu et al. proposed an interesting homogeneous electrochemical method that could avoid the interference of color and H_2_O_2_ for OPs analysis [85]. In addition, a white GeO_2_ nanozyme with only peroxidase-like activity was discovered by Liang and Han [68]. By coupling with AChE and ATCh, the GeO_2_ nanozyme could be utilized to realize the ultrasensitive and anti-interference detection of OPs based on the irreversible inhibition of AChE.

Another pathway of TCh changing the activity of nanozymes is to mask their active sites. For TCh, the sulfydryl group plays an important role in regulating the catalytic activity of nanozymes through the interactions between TCh and some metal atoms. For example, Li’s group developed a cobalt-graphene nanohybrid (Co-His-GQD-G) with excellent oxidase-like activity for the colorimetric detection of chlorpyrifos (Figure 3D) [73]. The cobalt nanoparticles dispersed on graphene sheets could be chelated by TCh produced from the hydrolysis of ATCh under the catalysis of AChE, blocking the catalytic active sites of Co-His-GQD-G for TMB oxidation.

As a matter of fact, multiple interactions between the sulfydryl group of TCh and nanozymes often coexist in a ChE-nanozyme cascade system [53,87,88]. Ni et al. developed a colorimetric sensing platform for AChE activity and its inhibitor based on peroxidase-like Prussian blue nanocubes, AChE, ATCh, H_2_O_2_ and TMB [87]. In their system, the generated TCh could cheat with active Fe^3+^ in the nanozyme, and it also chemically reduced the product TMBox to TMB again. Chen et al. prepared Co_3_O_4_@Co-Fe oxide double-shelled nanocages (DSNCs) with specific peroxidase-like activity for ChE detection [53]. In the AChE-Co_3_O_4_@Co-Fe oxide DSNCs system, TCh not only re-reduced TMBox to TMB, but also blocked the active sites of Co_3_O_4_@Co-Fe oxide DSNCs by coordinating with their metal centers.

#### 2.2.2. Generation of Other Compounds

**Figure 4:**
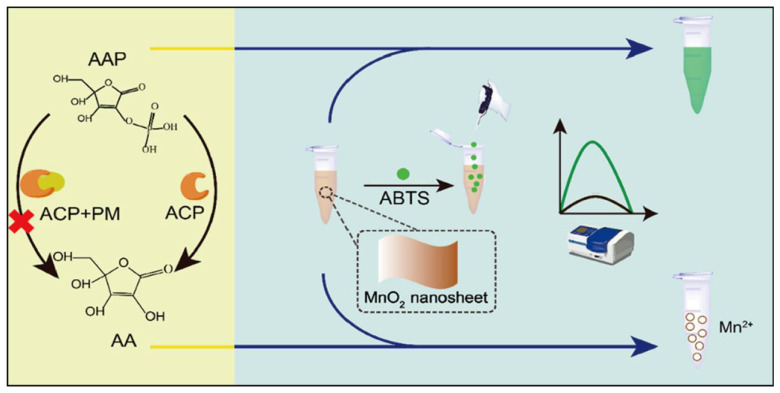
Generation of AA from ACP catalysis hinders nanozyme catalytic system for pesticide determination (reprinted with permission from [94], Copyright 2020, American Chemical Society.

In addition to thiol-containing TCh applied for OPs and biomarker sensing, some other compounds generated from enzyme catalysis can also enable OPs detection by affecting nanozyme-catalyzed reactions. For example, acid phosphatase (ACP) dephosphorylates ascorbic acid-2-phosphate (AAP) to ascorbic acid (AA), which can also induce the disintegration of MnO_2_ nanosheets. In the presence of OPs, ACP will lose its catalytic activity. Therefore, as displayed in Figure 4, Zhou’s group developed a sensitive colorimetric strategy based on the principle for the detection of ACP and its inhibitor [94]. Besides, Shah et al. found that Ch could result in the aggregation of cysteamine-capped gold nanoparticles (C-AuNPs) and weaken their ability to catalyze the oxidization of TMB [95]. Consequently, a C-AuNPs-based platform coupling with AChE was established to detect parathion-ethyl rapidly and sensitively.

Although the binary ChE-nanozyme assays have great potential for pesticide and related biomarker determination, they have some limits that will impede their commercial fabrication. For the generation of thiol-containing compounds, the interactions between nanozymes and thiol-containing compounds are principally based on the reducibility of the sulfhydryl group. Thus, other reducing substances possibly coexisting in real samples interfere greatly. For the generation of other compounds, the principle mainly depends on some specific enzymes and interactions, and the kind of pesticides that can be detected by the method is very limited.

## 3. Nanozyme-Only Assays

**Figure 5:**
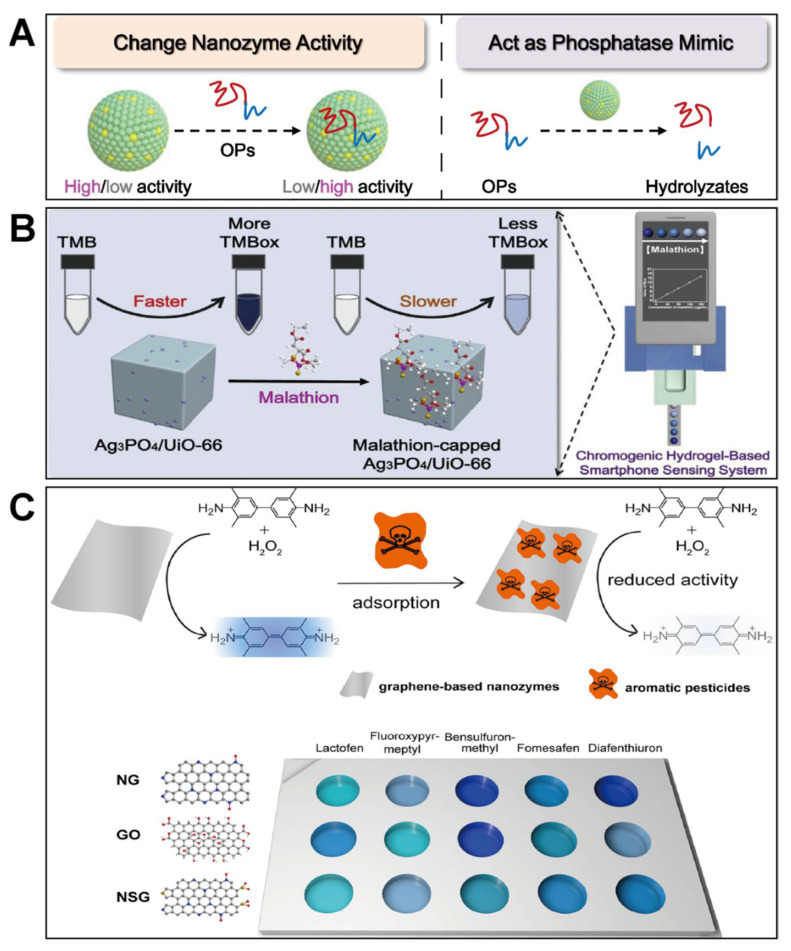
(**A**) shows nanozyme-only assays of OPs via changing nanozyme activity or using phosphatase mimics; (**B**) illustrates analyte-induced oxidase-mimicking activity loss of Ag_3_PO_4_/UiO-66 for colorimetric sensing of malathion (reprinted with permission from [19], Copyright 2021, Elsevier); (**C**) depicts a nanozyme-based colorimetric sensor array for discrimination of different pesticides (reprinted with permission from [99], Copyright 2020, American Chemical Society).

Apart from enzyme-like catalytic activity, nanomaterials often have some other properties. Therefore, some pesticide molecules can directly affect nanozyme activity or involve their catalytic reactions. With this consideration, some nanozyme-based sensors without the use of any bioenzymes were fabricated for detecting pesticides. According to the role of pesticides in these nanozyme-only sensors, they can be divided to the following categories (Figure 5A): on the one hand, pesticide molecules can induce the change of nanozyme activity through their specific interactions, and on the other hand, OPs will be hydrolyzed by phosphatase-mimicking nanozymes to produce signals.

### 3.1. Assays Based on Nanozyme Activity Modulation

For the assays based on nanozyme activity modulation, these nanozyme-only detection systems require that the target has unique structures and interactions with nanozymes [19,96,97,98,99,100,101,102]. For instance, our group developed a bioenzyme-free colorimetric assay of malathion, where the oxidase-mimetic activity of Ag_3_PO_4_/UiO-66 could be inhibited by the analyte by forming an Ag-S bond between the nanozyme and the pesticide [19]. Therefore, the target malathion could be detected by the TMB chromogenic reaction catalyzed by Ag_3_PO_4_/UiO-66 (Figure 5B). Similarly, PdAu rods and Au nanorods were also applied for the enzyme-free detection of pesticides based on forming the precious metal-S bonds [96,102]. Given most OPs contain the aromatic nucleus, Wei’s group fabricated a nanozyme-based colorimetric sensor array for the detection of pesticides based on the π-π stacking interaction and/or hydrogen bond between OPs and heteroatom-doped graphene [99]. When different aromatic pesticides were adsorbed onto the surface of heteroatom-doped graphene, they would mask the active sites of the nanozyme to various degrees. With the sensor array, different pesticides could be discriminated (Figure 5C).

### 3.2. Assays Based on Phosphatase-like Nanozymes

Except for the common peroxidase- and oxidase-like catalytic activities of nanozymes for pesticide detection, nanozymes with phosphatase-like activity were also explored, which can destroy the phosphate ester bond in OPs. In these assays, OPs are not only the analyte, but also act as a reagent to generate signals. The representative phosphatase-like nanozymes include Ce-based [103,104,105] and Zr-based materials [106,107,108]. For example, to realize the determination of pesticides, Wu et al. prepared a ZrO_2_/CeO_2_/PAA nanocomposite with remarkable phosphatase-mimicking activity, which could degrade methyl-paraoxon to *p*-nitrophenol with a yellow color for colorimetric detection [105]. By using the inner filter effect between *p*-nitrophenol and carbon nanodots, the target methyl-paraoxon could also be detected by a fluorescence method [104]. Besides, by monitoring the oxidation current of *p*-nitrophenol produced from the hydrolysis of methyl-paraoxon catalyzed by phosphatase-mimicking CeO_2_, Sun et al. realized the electrochemical determination of the target efficiently [103].

Obviously, one of the attractive advantages of these nanozyme-only biosensors is their excellent tolerant to harsh environments, which should be attributed to the high stability of nanozymes. However, it is difficult to discover the special interactions between nanozymes and targets for their sensing, greatly restricting the development of such systems. Furthermore, different from ChE as a selective recognition unit, selectivity is a major problem for these nanozyme-only detection platforms.

## 4. Nanozyme-Based Immunosensors

**Figure 6:**
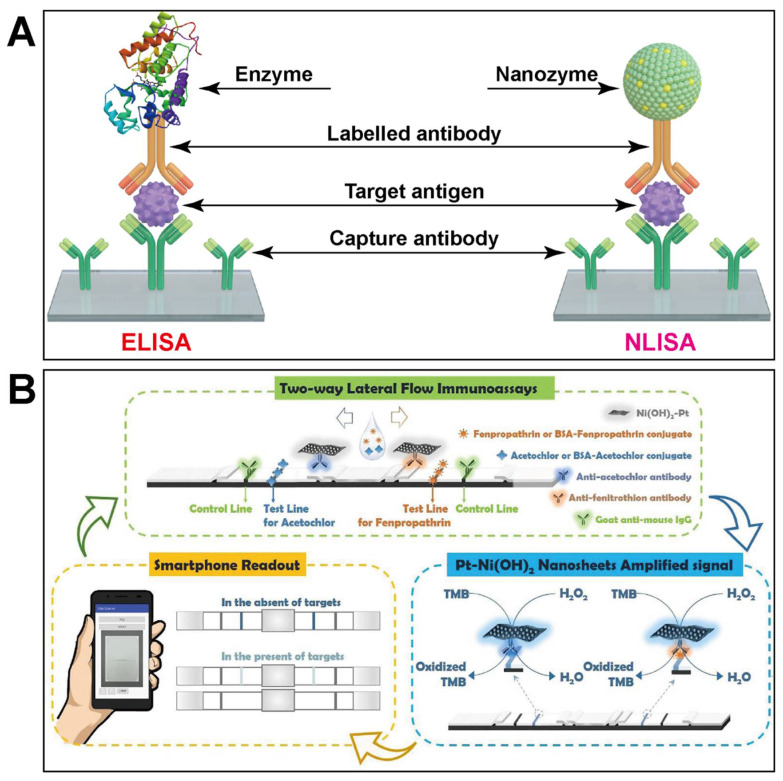
(**A**) compares NLISA and conventional ELISA with a sandwich-like configuration; (**B**) illustrates design of a nanozyme-based two-way lateral flow immunoassay for pesticide biosensing (reprinted with permission from [111], Copyright 2019, Elsevier).

Immunoassays are high-selectivity and high-throughput analytical tools based on the specific recognition of antibodies and antigens [109,110]. In conventional enzyme-linked immunosorbent assays (ELISA), HRP or other bioenzymes are usually labelled on an antibody to realize the immunosensing of pesticides. Owning to excellent stability and enzyme-mimicking activity, some nanozymes can replaced the bioenzymes applied in immunoassays. Therefore, typical nanozyme-linked immunoassays (NLISA) were established by replacing HRP with a peroxidase-mimicking nanomaterial. Figure 6A compares representative sandwich-type ELISA and NLISA. As illustrated, the primary antibody is immobilized on the surface of 96-well plates, which can selectively capture the target antigen. Then, the nanozyme-labelled secondary antibody can recognize the antigen to form a sandwich-like configuration. The conjugated nanozyme can catalyze a chromogenic reaction to amplify signals with high sensitivity, where the signal intensity is closely related to the amount of the target antigen captured by the nanozyme-labeled secondary antibody and the primary antibody simultaneously. Such double specific identification for the target antigen endows NLISA with excellent selectivity. Besides, nanozymes are supposed to be more stable and have a lower cost. Therefore, more and more NLISA are being fabricated for the quantification of pesticides [111,112,113,114,115].

Typically, Du’s group proposed a two-way lateral flow immunoassay (LFI) for the simultaneous detection of two pesticides based on nanozyme-catalyzed signal amplification [111]. As shown in Figure 6B, both the antiacetochlor antibody and the antifenitrothion antibody were labelled by 2D Pt-Ni(OH)_2_ NSs with high peroxidase-like activity. The difference between positive and negative test lines could be amplified by the catalytic reaction between Pt-Ni(OH)_2_ NSs and the TMB/H_2_O_2_ mixture. Therefore, the explored NLISA could achieve high sensitivity and wide detection range for the two pesticides via the nanozyme-based signal enhancement strategy [111].

Although NLISA show many merits in comparison with bioenzyme-participated immunoassays in terms of stability and adjustability, some challenges exist for their commercial fabrication and use. Firstly, the antibodies available for different pesticides are still very limited. Besides, the means used to conjugate nanozymes with antibodies are still in the laboratory stage. Finally, with no standardization to follow, these reported nanozyme labels and immunoassays are currently under construction in an individual lab as a proof-of-concept. As a promising detection method, more efforts are needed to promote the standardization and industrialization of NLISA.

## 5. Nanozyme-Based Aptasensors

**Figure 7:**
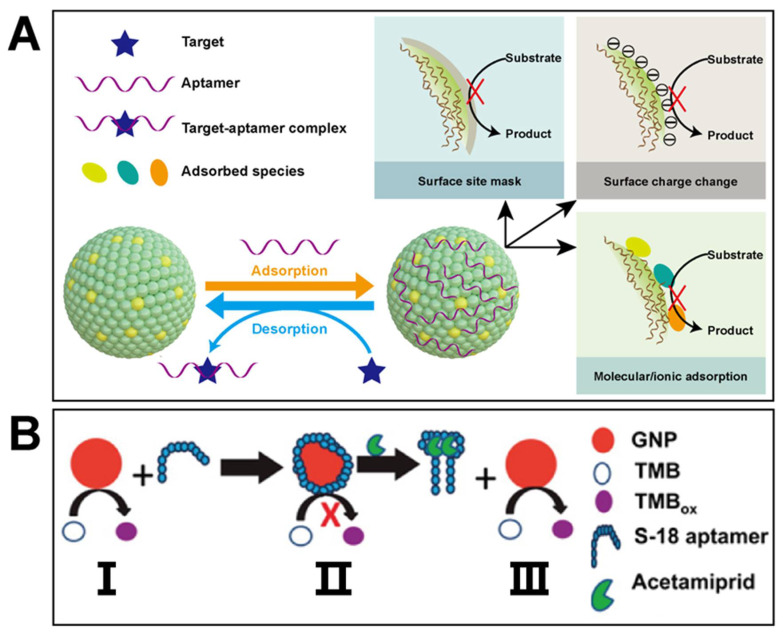
(**A**) presents potential impacts of aptamers on catalytic performance of nanozymes; (**B**) shows principle of detecting acetamiprid using a gold-based nanozyme and a specific aptamer (reprinted with permission from [122], Copyright 2014, American Chemical Society).

Aptamers are defined as short sequences of DNA with strong and specific affinity toward target molecules. Thanks to the systematic evolution of ligands by exponential enrichment (SELEX) technique developed and applied to screen aptamers efficiently, they are widely used in the building of biosensors [116,117]. Aptamers can change the inherent nature of nanozymes through the special interactions originating from the structural and chemical characteristics of aptamers [28]. Therefore, combing nanozymes with aptamers is an effective approach to obtain high sensitivity and good selectivity at the same time. Nowadays, using aptamers as recognition elements and integrating them with nanozymes became a hot topic for various target sensing. Similar to DNA chains [118,119,120,121], aptamers can adsorb onto nanozyme surface to adjust the catalytic feature of these nanozymes in different behaviors (Figure 7A): (1) the activity sites on nanozyme surface can be masked by the adsorption of aptamers, which may weaken the catalytic activity of nanozymes; (2) the adsorbed aptamers can change the surface charge of nanozymes, which affects the adsorption kinetics of substrates via electrostatic interaction; (3) the adsorbed aptamers on nanozymes may further lead to the adsorption of other ions and molecules, which can also change the catalytic performance of nanozymes. When the target appears, the corresponding aptamer is specifically bounded by the target and desorbed from nanozyme surface. As a result, the enzyme-like activity of nanozymes is restored. All the behaviors mentioned above enable the construction of aptamer-nanozyme sensors to achieve the qualitative and quantitative detection of pesticides and biomarkers.

Typically, Weerathunge et al. reported a rapid, highly specific, and sensitive approach to detect a neurotoxic pesticide by combining peroxidase-mimetic gold nanoparticles (GNPs) with an acetamiprid-specific S-18 aptamer (Figure 7B) [122]. They used the target-specific ssDNA aptamer to inhibit the peroxidase-like activity of GNPs through the adsorption between the aptamer and GNPs. In the presence of the target pesticide, the aptamer would leave the surface of GNPs in a target-responsive structural change manner. Subsequently, the peroxidase-like activity of GNPs resulted in the colorless TMB oxidation to blue TMBox. Because of the specificity of aptamers and the sensitivity of GNPs, the proposed platform exhibited at least 5-fold more sensitivity and was 3-times faster than that of the previously reported methods. In their another study, a chlorpyrifos aptamer was screened out to turn on/off the peroxidase-mimic activity of tyrosine-capped silver nanoparticles, which could further be used for the detection of chlorpyrifos [123].

Nanozyme-based aptasensors are meaningful tools to achieve the specific detection of pesticides. In these systems, both nanozymes and aptamers are more stable and cheaper than enzymatic sensing materials like AChE and CHO. Aptamers act as a bridge linking the target to nanozymes, and the intrinsic catalytic activity of nanozymes endows the aptamer recognition process with amplified signals for quantitative analysis. The two complements make nanozyme-based aptasensors have great promise in pesticide detection. However, as a prerequisite to achieve the sensitive and selective detection of pesticides, the efficiency of the SELEX process to screen aptamers with high selectivity still needs to be improved. Besides, the interactions between nanozymes and aptamers are complicated, which still need to be investigated thoroughly. Also, exploring how to enhance the conjugation and adsorption/desorption behaviors of aptamers will be beneficial to regulate the detection performance purposely.

## 6. Conclusions and Perspectives

The huge threat posed by pesticide residues to human health and the environment attracted abundant intention and interest in developing efficient methods and tools for their monitoring. Undoubtedly, nanozymes with the merits of low costs, easy large-scale production, excellent tolerance to harsh conditions, long-term storability, and on-demand tailorable performance found potential use in the field. In this review, recent developments of nanozyme-involved biosensors for pesticide and related biomarker detection were highlighted. The increasing number of publications indicates that this field is experiencing a period of rapid growth [124,125]. By integrating nanozymes with other recognition elements (bioenzymes, aptamers, antibodies/antigens, et al.), several kinds of biosensors with good performance were explored for pesticide and ChE sensing. With phosphatase-mimicking nanozymes as the only sensing element, successful detection of targets with amplified signals was also realized. Although these advances were made, some challenges remain in the emerging area, and further efforts are required to advance nanozyme-based pesticide sensors for practical applications:

(1) Currently, most pesticide detection processes are based on bioenzyme–nanozyme integrated assays. In ternary or binary cascade systems, natural enzymes like ChE are still necessary to act as a recognition unit, and nanozymes can only replace a part of these bioenzymes (e.g., HRP). Given that natural enzymes with frail activity are still used in these assays, the vulnerability against harsh conditions makes them face great challenges during real applications. To solve the issue, an efficient way is to immobilize bioenzymes and nanozymes together onto appropriate matrices [65,126], where both high-efficiency tandem catalytic performance and satisfied stability can be obtained for target detection. Another solution is to explore novel nanozymes that can completely replace ChE and CHO. Once this aim is realized, cascade catalytic systems composed of all-inorganic nanomaterials can be fabricated for pesticide sensing with robust performance.
(2) Another efficient strategy for pesticide and ChE biosensing is to combine nanozymes with other elements like aptamers and antibodies/antigens. In these assays, appropriate conjugation of inorganic nanozymes and biological molecules is crucial because the bioconjugation efficiency often determines its sensing performance. More approaches to immobilize antibodies or aptamers onto nanozyme surface are needed to produce robust and controllable conjugates. Additionally, since the interactions between nanozymes and some biological elements like aptamers are quite complex, the underlying mechanisms are still not fully uncovered and understood [28], affecting the design and fabrication of new biosensors for target analysis. For better detection, more efforts should be focused on studying the generation of interactions and the elimination of side effects.
(3) Direct interaction of nanozymes with targets is an effective means to detect pesticides, which can avoid the negative effects from biological substances. However, the nanozyme-only systems developed currently are limited to phosphatase-like nanozymes and specific pesticide molecules. On the one hand, the design and development of nanozymes with only phosphatase-like activity remain a challenge, and the substrates available are also very limited. On the other hand, the interactions between pesticides and nanozymes are complicated and vulnerable, which are easily disturbed by external conditions. More importantly, given that no identification units are involved, selectivity is a major problem in such a method. A possible pathway to improve the detection selectivity is to fabricate nanozyme-based sensor arrays for the identification and differentiation of multiple targets [99,127,128,129]. Besides, the molecular imprinting technique can also be introduced to fabricate molecularly imprinted nanozymes for specific catalysis and sensing [130,131].
(4) According to the examples mentioned above, most of nanozyme-participated systems for pesticide and ChE sensing are based on the colorimetric mode. Although such a mode shows the advantages of easy signal reading and result visualization, poor sensitivity may limit its use in low-content target analysis. More advanced techniques (fluorescence, electrochemical, photoelectrochemical, SERS, et al.) are expected to be combined with nanozyme catalysis for better detection. For instance, some products originating from nanozyme catalysis can interact with additional species with the fluorescence feature, and they can promote or quench the luminescence of the latter via inner filter effect, photoinduced electron transfer, Förster resonance energy transfer, aggregation induced emission, intramolecular charge transfer, and so on, providing the basis for sensing targets using the fluorescence mode with higher sensitivity [81,82]. Besides, the species produced from nanozyme catalysis as well as the substrates used for nanozyme catalysis can also be probed by electrochemistry via recoding their redox signals [85,132], enabling us to monitor analytes via electrochemical devices. Apart from conventional single-mode detection, multimode sensing strategies with inherent self-correction and self-calibration features can also be developed for pesticide and related biomarker determination [33].
(5) Although a number of nanozyme-participated methods were explored and verified in lab for pesticide and ChE sensing, their feasibility in real environments still needs validation. For practical applications, complex matrices and environments may make non-negligible impacts on the monitoring of targets in terms of specificity, sensitivity, and accuracy. This requires more verification tests performed in real scenarios to promote the practicability of these sensors. On the other hand, standardized fabrication and quality control of these pesticide biosensors are critical. Apart from the standardization of nanozymes [133], the manufacturing process of nanozyme-based sensors should also be normalized before promoting them to commercial production and use.

## Figures and Tables

**Table 1:** Nanozyme-involved detection of pesticides and cholinesterases based on different mechanisms caused by the generation of thiol-containing compounds.

Analyte	Nanozyme	Activity	Detection Mode	Mechanism	Detection Range	Detection Limit	Reference
Dichlorvos; Methyl-paraoxon	PAA-CeO_2_	Oxidase	Colorimetric	Competitive oxidation	–	8.62 ppb; 26.73 ppb	[74]
AChE	CoN_x_-NC	Oxidase	Colorimetric	Competitive oxidation	0.6–800 mU/L	0.2 mU/L	[75]
AChE	PdSP@rGO	Oxidase	Colorimetric	Competitive oxidation	0.25–5 mU/mL	0.0625 mU/mL	[76]
AChE; Paraoxon-ethyl	MIL-101(Fe)	Peroxidase	Colorimetric	Competitive oxidation	0.2–50 mU/mL; 8–800 ng/mL	0.14 mU/mL; 1 ng/mL	[77]
Carbaryl	NH_2_-MIL-101(Fe)	Peroxidase	Ratiometric fluorescence	Competitive oxidation	2–100 ng/mL	1.45 ng/mL	[31]
AChE	Fe-N-C SAC	Oxidase	Colorimetric	Competitive oxidation	0.1–25 mU/mL	0.014 mU/mL	[78]
Chlorpyrifos	CeGONRs	Oxidase	Colorimetric	Competitive oxidation	0.012–3.50 μg/mL	3.43 ng/mL	[79]
AChE; Paraoxon-ethyl	Fe^3+^:MOFs/TiO_2_NM	Peroxidase	Electrochemical	Competitive oxidation	0.01–100 mU/mL; 0.01–5.0 μg/mL	0.01 mU/mL	[80]
AChE	Fe/NPC	Oxidase	Fluorescence; Colorimetric	Competitive oxidation	0.02–5.0 U/L; 0.01–5.0 U/L	0.0032 U/L; 0.0073 U/L	[81]
BChE	Fe-N-C SAN	Peroxidase	Colorimetric	Competitive oxidation	0.1–10 U/L	0.054 U/L	[32]
AChE	Fe-SAs/NC	Peroxidase	Ratiometric fluorescence	Competitive oxidation	2–70 U/L	0.56 U/L	[82]
Paraoxon	GeO_2_	Peroxidase	Colorimetric	Decomposing nanozyme	0.1–50 pM	14 fM	[68]
AChE; Omethoate; Dichlorvos	γ-MnOOH NWs	Oxidase	Colorimetric	Decomposing nanozyme	0.01–1.25 mU/mL; 5–50 ng/mL; 1–10 ng/mL	0.007 mU/mL; 0.35 ng/mL; 0.14 ng/mL	[83]
AChE; Paraoxon	MnO_2_ nanosheets	Oxidase	Colorimetric	Decomposing nanozyme	0.1–15 mU/mL; 0.001–0.1 μg/mL	35 μU/mL; 1.0 ng/mL	[72]
AChE	MnO_2_ nanosheets	Oxidase	Colorimetric	Decomposing nanozyme	25–500 mU/mL	0.18 mU/mL	[84]
Paraoxon	MnO_2_ sheets	Oxidase	Electrochemical	Decomposing nanozyme	0.1–20 ng/mL	0.025 ng/mL	[85]
Malathion	Cu^2+^-g-C_3_N_4_	Peroxidase	Fluorescence; Colorimetric	Masking active sites	70–800 nM; 2.5–25 nM	6.798 nM; 1.204 nM	[86]
Chlorpyrifos	Co-His-GQD-G	Oxidase	Colorimetric	Masking active sites	2–20 ng/mL	0.57 ng/mL	[73]
AChE	PB NCs	Peroxidase	Colorimetric	Multiple mechanisms	0.1–5.0 mU/mL	0.04 mU/mL	[87]
Chlorpyrifos	CeO_2_ NPs	Oxidase	Colorimetric	Multiple mechanisms	50–1000 ng/mL	7.6 ng/mL	[88]

## Abbreviations

2-HTA	2-Hydroxy terephthalic acid
AA	Ascorbic acid
AAP	Ascorbic acid-2-phosphate
ABTS	2,2-Azobis(3-ethylbenzothiazolin-6-sulfonic acid)
ACh	Acetylcholine
AChE	Acetylcholinesterase
ACP	Acid phosphatase
ATCh	Acetylthiocholine
AuNPs/MOG(Fe)	Fe-based metal–organic gel supported gold nanoparticles
BChE	Butylcholinesterase
C-AuNPs	Cysteamine-capped gold nanoparticles
Ch	Choline
ChE	Cholinesterases
CHO	Choline oxidase
CoN_x_-NC	N-doped carbon nanocages containing Co–N_x_ active sites
CPs	Carbamate pesticides
DAB	3,3′-Diaminobenzidine
DPA	Diaminophenazine
DSNCs	Double-shelled nanocages
ELISA	Enzyme-linked immunosorbent assays
Fe/NPC	N-doped hierarchical porous carbon-supported iron
Fe-SAs/NC	Single-atom iron anchored on N-doped porous carbon
GNPs	Gold nanoparticles
HRP	Horseradish peroxidase
LFI	Lateral flow immunoassay
MNPs	Magnetic nanoparticles
NH_2_-MIL-101(Fe)	Fe-based metal–organic frameworks
NLISA	Nanozyme-linked immunoassays
NWs	Nanowires
OPD	*o*-Phenylenediamine
OPs	Organophosphorus pesticides
PAA-CeO_2_	Polyacrylic acid-coated cerium oxide nanoparticles
PB NCs	Prussian blue nanocubes
PdSP@rGO	Pd square nanoplates grown on reduced graphene oxide
SAC	Single-atom catalysts
SAN	Single-atom nanozymes
SELEX	Systematic evolution of ligands by exponential enrichment
SERS	Surface enhancement Raman spectroscopy
TA	Terephthalic acid
TCh	Thiocholine
TMB	3,3′,5,5′-Tetramethylbenzidine
